# The Role of Metacognition in the Prediction of Depressive and Anxiety Symptoms in Chronically Ill Patients

**DOI:** 10.3390/jcm13051306

**Published:** 2024-02-25

**Authors:** Agata Kołodziejczyk, Julia Krawczyk, Elżbieta Tkaczyszyn-Mika, Julia Gniewczyńska, Michał Ziarko, Dorota Zozulińska-Ziółkiewicz, Tomasz Pawłowski

**Affiliations:** 1 Division of Psychotherapy and Psychosomatic Medicine, Department of Psychiatry, Wroclaw Medical University, 50-367 Wrocław, Poland; 2 Lower Silesian Oncology, Pulmonology and Hematology Center, 53-413 Wroclaw, Poland; 3 Department of Health and Clinical Psychology, University of Adam Mickiewicz, 60-568 Poznan, Poland; 4 Department and Clinic of Internal Medicine and Diabetology, Medical University of Karol Marcinkowski, 61-701 Poznan, Poland; 5 University Teaching Hospital, 65-046 Zielona Góra, Poland

**Keywords:** anxiety, chronic illness, depression, metacognition, metacognitive beliefs

## Abstract

Introduction: Cancer, diabetes, and heart diseases are frequent causes of depression and anxiety. The study explored the metacognitive beliefs manifested by chronically ill patients and the presence of depressive or anxiety symptoms and the predictive role of metacognition in both. Methods: A total of 254 chronically ill patients participated in the study. The Metacognitive Questionnaire was used to measure the patients’ metacognitive beliefs, whereas the Hospital Anxiety and Depression Scale was applied to evaluate their psychopathological symptoms. A correlation analysis was performed to explore the relationships between metacognition and psychopathological symptoms. Regression analyses were conducted to examine the predictive role of metacognition in anxiety and depression. Results: The Negative Beliefs about Uncontrollability and Danger scale correlated with both anxiety and depression scales, and the Cognitive Confidence scale correlated with the depression scale. Linear regression analyses indicated that metacognitive beliefs were responsible for 32.2% of the variance of anxiety symptoms among all the chronically ill. Metacognitive beliefs accounted for 48.8% of the variance in anxiety symptoms and 36.6% in depressive symptoms among diabetes patients. Conclusions: There are specific correlations between psychopathological symptoms and metacognition among chronically ill patients. Metacognitions have a moderate role in developing and sustaining anxiety and depressive symptoms.

## 1. Introduction

The World Health Organization (WHO) reports that the diseases which are most common and whose contribution to the global mortality rates is the highest include the following: cardiovascular diseases, cancer, chronic respiratory failures, and type II diabetes (T2DM). Apart from the deterioration of the physical functioning of patients suffering from them, all mentioned illnesses also affect their mental state and psychological well-being [1,2]. Depressive and anxiety symptoms in chronic diseases are among the most manifested psychopathological symptoms [3]. Their impact on the patients’ lives is multifaceted. They significantly reduce the quality of life, deteriorating everyday functioning, as well as decreasing satisfaction with professional and/or sexual life and damaging personal relationships [4]. Additionally, they contribute to a more significant burden on the health service and translate into a later return to patients’ professional activity, to name just a few consequences [3,5]. Among the diseases listed by the WHO, depressive and anxiety symptoms and their negative impact on the patients’ lives have been undoubtedly confirmed in the group of cancer patients [6] and those suffering from heart disease and type II diabetes [7]. Therefore, scientists worldwide have launched research on identifying factors that may influence development of depression and anxiety symptoms among chronically ill patients to design targeted therapeutic solutions to reduce the occurrence of anxiety and depression among them. World Health Organization (WHO), WHO Global NCD Action Plan 2013–2020, 2013, WHO; Geneva, Retrieved from: https://www.who.int/nmh/events/ncd_action_plan/en/ (accessed on 1 January 2020.). British Heart Foundation, The National Audit of Cardiac Rehabilitation Annual Statistical Report, Retrieved from: https://www.bhf.org.uk/informationsupport/publications/statistics/national-audit-of-cardiacrehabilitation-annual-statisticalreport-2017, 2017 (accessed on 1 January 2020).

Among the researchers who took up this issue were Wells and Matthews [8], who put forward a hypothesis about the influence of cognitive meta-beliefs on the formation of depressive and anxiety disorders. The authors define metacognitive beliefs as “the aspect of information processing that monitors, interprets, evaluates and regulates the contents and processes of its organization” [9]. According to the authors, vulnerability towards and maintenance of psychopathological symptoms are directly related to a non-specific style of thinking called Cognitive Attentional Syndrome (CAS). CAS consists of repetitive negative thinking within worrying and ruminating processes [6]. The decisive factors for launching CAS are negative and positive beliefs regarding the processes mentioned above. According to Wells, negative meta-beliefs are factors that generate and maintain mental disorders [9]. The authors developed a metacognitive theory of mental disorders and a questionnaire for their research (Metacognitive Questionnaire MCQ-30) [10].

Since the development of Wells’ theory, numerous studies have proven the role of cognitive meta-beliefs in the development and maintenance of psychopathological symptoms [3,5], including psychiatric disorders such as general anxiety disorder (GAD), obsessive-compulsive disorder (OCD), depression and post-traumatic stress disorder (PTSD) [9], and somatic issues [11]. It has been recognized that cognitive meta-beliefs influence the persistence of negative emotions [12] and play a significant role in coping with stress [13], which is important in fighting a chronic disease. Consequently, numerous studies are still being carried out to determine the nature of the relationship between cognitive meta-beliefs and the development and maintenance of depressive and anxiety symptoms in groups of patients suffering from chronic somatic diseases. So far, such investigations have been pursued with regards to patients suffering from Parkinson’s disease [14,15], epilepsy [16], cancer [5,6,17,18], diabetes [7,19,20,21], chronic fatigue syndrome [22,23], and HIV [24]. All these studies have confirmed the correlation between cognitive meta-beliefs and psychopathological symptoms. However, what draws attention is the deficiency of studies in the group of patients suffering from cardiovascular diseases [25]. Studies increasingly focus on the correlation between the predictive nature of certain negative cognitive meta-beliefs and the emergence and maintenance of depressive and anxiety symptoms. Such studies are most often conducted in homogeneous groups representing a given disease entity. Single studies comparing two groups of patients are available [3,18,26]. However, the authors of the present study have not found examples of studies which compare the predictive and sustaining role of cognitive meta-beliefs in as many as three groups of diseases. Considering the WHO data, comparisons between cancer patients and diabetics and those suffering from a cardiovascular disease seemed even more interesting and advisable.

Therefore, this study aims to check the relationship between cognitive meta-beliefs and depressive and anxiety symptoms in three groups of patients with chronic diseases, cancer, type II diabetes, and cardiovascular diseases, and the predictive nature of cognitive meta-beliefs in specific psychopathological symptoms. The research also attempts to examine the correlation of cognitive meta-beliefs with depressive and anxiety symptoms and their predictive nature for these symptoms among the studied patients.

## 2. Materials and Methods

### 2.1. Participants

A total of 254 patients participated in the research. The study included 110 oncological, 40 cardio, and 104 T2DM patients. Each group was recruited in a different medical center specific to the mentioned illnesses. Oncological patients were treated at the Lower Silesian Oncology Centre in Wrocław. A sample of T2DM patients was recruited at the Department and Clinic of Internal Medicine and Diabetology in Poznan. Finally, participants with cardiovascular conditions were being treated at the University Teaching Hospital in Zielona Góra. All patients were native Polish speakers and Polish nationals. The sociodemographic information for the researched sample is presented in Table 1. The sample consisted of 135 women and 119 men, including 79 women diagnosed with cancer, 11 being treated for cardiovascular diseases, and 45 diabetics and 31 men diagnosed with cancer, 29 cardio patients, and 59 male diabetics (demographic data are presented in Table 1). Other demographics concerned age, education, marital status, and place of residence. All mentioned factors have been proven to be mediators or predictors of psychological well-being in chronically ill patients. The study followed the regulations of the Declaration of Helsinki and was approved by the Ethics Committee of Wrocław Medical University (no. 820/2021). Informed consent was obtained from all subjects involved in the study.

### 2.2. Measurements

The patients who participated in the study were asked to fill in two questionnaires—the Hospital Anxiety and Depression Scale (HADS) and the Metacognitive Questionnaire (MCQ)—as well as a sociodemographic questionnaire created specifically for the study.

The Polish adaptation of the Metacognitive Questionnaire (MCQ) by Adrian Wells and Samantha Cartwright-Hatton was used to study metacognitive beliefs [10]. The Polish version’s mean Cronbach’s alpha is 0.79 [27]. The questionnaire consists of 65 questions. Patients respond about their meta-beliefs using a 4-point Likert scale, where 1 means “disagree” and 4 “completely agree”. The results are obtained by summing the answers given by the patients on the following five scales:

- Positive beliefs about worry;
- Negative beliefs about uncontrollability and danger;
- (Lack of) cognitive confidence;
- Need to control thoughts;
- Cognitive self-consciousness.

The Hospital Anxiety and Depression Scale (HADS) was used to test depressive and anxiety symptoms. The mean Cronbach’s alpha for HADS-A is 0.83 and for HADS-D is 0.82 [28]. The questionnaire is completed by answering 14 questions, 7 each in anxiety and depressive symptom groups. The score is obtained by summing up the patient’s responses. The level of results from which it should be considered that the patient manifests psychopathological symptoms at the non-adaptive level and requires intervention is still debatable. However, a result above 7 (out of a possible 21) on any of the scales suggests a significant level of psychopathology.

The sociodemographic questionnaire created for the study recorded answers on gender, age, education, marital status, and place of residence.

### 2.3. Statistical Analysis

Statistical analyses were performed using the Statistica program. The r-Pearson correlations were performed to examine the relationship of cognitive meta-beliefs with depressive and anxiety symptoms among patients representing the three mentioned chronic diseases. Linear regression analyses were performed to test the predictive role of cognitive meta-beliefs in the development and maintenance of psychopathological symptoms. Data used in the conducted research will be available upon request.

## 3. Results

### 3.1. r-Pearson Correlations

Two statistically significant correlations between MCQ and HADS results were found regarding cancer patients. The negative beliefs and the need to control thoughts scales (MCQ) were strongly correlated with the anxiety scale (HADS) (Table 2).

There were three statistically significant correlations regarding HADS and MCQ scores in cardio patients. The negative beliefs, the cognitive confidence, and the cognitive self-consciousness scales (MCQ) were strongly correlated with the anxiety scale (HADS) (Table 3).

As many as eight statistically significant correlations between MCQ and HADS questionnaire scores were found in T2DM patients. The positive beliefs, the negative beliefs, and the cognitive confidence scales (MCQ) correlated with anxiety and depression scales (HADS). Moreover, the need to control thoughts and the cognitive self-consciousness scales (MCQ) correlated with the anxiety scale (HADS) (Table 4).

Finally, three statistically significant results were found regarding all studied chronically ill patients. The negative beliefs scale (MCQ) correlated with both the anxiety and depression scale (HADS). Furthermore, the cognitive confidence scale (MCQ) correlated with the depression scale (HADS) (Table 5).

### 3.2. Linear Regression Analyses

Linear regression analyses demonstrated that metacognitive beliefs were responsible for 32.2% of the variance of anxiety symptoms among all tested patients. Moreover, metacognitive beliefs accounted for 48.8% of the variance in anxiety symptoms and 36.6% in depressive symptoms among T2DM patients. All other results were not statistically significant, and, for that reason, Table 6 presents only those models which showed a predictive value of metacognitions in anxiety and depressive symptoms.

## 4. Discussion

The present study is the first to examine the relationship between cognitive meta-beliefs and psychopathological symptoms among the following three categories of chronic conditions: oncological, cardiological, and T2DM. The results have yielded some interesting insights.

The data point out that metacognitive beliefs may be related to anxiety and depression among chronically ill patients (considering the whole studied group). The same evidence was provided before in homogeneous and heterogeneous chronically ill patient groups [14,15,16,17]. Those data address the overall support for the validity of the role of metacognition in anxiety and depression development and its persistence across many chronic conditions.

Moreover, across all three conditions, the negative beliefs scale presents a factor strongly associated with anxiety, which resembles Wells’ CAS theory. Specialists working clinically with patients with chronic diseases should be mindful of the CAS high-risk group. The diagnosis of anxiety disorders should encourage specialists to look for the causes of this state in recurrent negative thoughts and to consider addressing this issue with Metacognitive Therapy (MCT). According to the meta-analysis prepared by Normann and Morina [29], MCT has proven to be an effective form of therapy in a wide range of mental disorders, especially depression and anxiety. Furthermore, even though this field of study is yet to be developed, several studies have already provided data on the beneficial impact of MCT, for example, in a group consisting of oncological patients [30,31]. Consequently, the notion that chronically ill patients diagnosed with CAS can benefit from this approach seems viable.

In addition to similarities to the previous research, interesting differences were found between these groups when the oncological, cardio, and T2DM patients were considered separately. For example, the research examined meta-beliefs in the group of cardiological patients [32], rarely studied in that regard. Data revealed that cognitive confidence and self-consciousness are correlated with anxiety, apart from negative beliefs, in this group. Cognitive confidence in the literature is described as one’s opinion about one’s poor cognitive abilities, i.e., memory, whereas self-consciousness is a term to describe the tendency to observe one’s cognitions [25]. Wells et al. [33] justify this mechanism as an effect of giving up on active coping in the face of stress in a group of overly self-conscious people. Negative beliefs, negative assessment of one’s cognitive abilities, and too intense self-focus might present an anxiety receptivity in the cardiac disease patient group, but that hypothesis needs more support. Unfortunately, in the group of patients suffering from heart disease, anxiety disorders can exacerbate the disease process and increase mortality [34]. The inclusion of psychotherapy in the cardiac group of patients with CAS would be an appropriate step to improve the quality of life and boost treatment outcomes. A recent study about MCT application among cardiac patients with a 12-month follow-up also presented satisfactory results in reducing anxiety and depression compared to standard group-based programs [35].

On the other hand, data concerning the oncology group of patients presented findings that demonstrated correlations between metacognitive beliefs, particularly negative beliefs about uncontrollability and danger, and the need to control thoughts and anxiety symptoms. These results are coherent with multiple studies, reinforcing the idea of the strong impact of metacognition on psychological well-being among oncological patients. Negative beliefs and the need to control thoughts and positive beliefs about worry and (lack of) cognitive confidence were found to be factors accounting for predicting and maintaining anxiety and depressive symptoms in previous studies [6,26]. Furthermore, metacognitive beliefs were also found to be responsible for post-traumatic stress disorder symptoms in the oncological population [5,36], providing strong evidence of the importance of metacognitive diagnosis and treatment in the mentioned group for maintaining psychological well-being in the event of prolonged and trying times of oncological treatment. However, it is essential to highlight that, unlike many other studies on similar matters, the present study has provided evidence on the correlations of metacognitive beliefs with psychopathological symptoms within the oncological group but not the predictive value of metacognitive beliefs within them. The reason for that might be the statistically low intensity of anxiety and depressive symptoms in the tested group (M = 7.48 and M = 6.48, accordingly) by the time the study was conducted. Notwithstanding, the presented correlations support the need to continue research towards describing the connection between metacognitive beliefs and the psychological state of oncological patients to develop more effective paths of psychosocial support during and after treatment.

Finally, an interesting side finding was developed from the group with T2DM. Diabetes patients had the strongest correlations between metacognitions and depression and anxiety, apart from other study groups. A possible reason for this discrepancy might be that all these conditions have various symptomatology, so they affect patients differently. However, that hypothesis needs further support. What also seems insightful is that, unlike previous studies on a group of people with diabetes [19,20,21], this study showed surprisingly numerous correlations between multiple levels of meta-beliefs and depression and anxiety. Perhaps it is related to the selection of the research group, i.e., individuals undergoing inpatient treatment. The literature shows that diabetic patients develop symptoms of depression and anxiety during hospitalization due to unfamiliar environments and exposure to a lack of intimacy [37]. Nevertheless, the research lacks the data that would sufficiently support this hypothesis.

The presented study has some limitations that need addressing. Compared with T2DM and oncological patients, the cardio group was visibly underrepresented, which might have impacted the validity and reliability of the study. Another issue is lack of randomization, which was impossible for ethical reasons. The patients were invited to participate in the study regardless of their course of treatment in every specific group. Lack of randomization could undoubtedly affect the results through the uneven distribution of different clinical and individual factors. However, the purpose of this study was to uncover specific differences between different chronic conditions; so, in this light, the mentioned lack of randomization seems more validated.

Last but not least, the study did not control for either psychiatric medication intake among the tested patients or any previous psychiatric disorders which could influence levels of depression and anxiety symptoms and which cannot be excluded as confounding factors since chronically ill patients turn to psychiatric help quite often. This also includes other medication that can lead to mood changes, e.g., statins in cardiovascular diseases that can lower one’s mood. This should be considered in studies to come. It also seems relevant to consider marital status as another factor or to attempt to balance the compared groups in terms of marital status in order to provide better insight into its influence on the patients’ well-being, especially given that marital status and a partner’s support are often responsible for a patient’s psychosocial well-being.

## 5. Conclusions

This study explored the link between cognitive meta-beliefs and both depressive and anxiety symptoms in three groups of patients with the following chronic diseases: cancer, T2DM, and cardiovascular diseases. What is more, it assessed the predictive nature of cognitive meta-beliefs in the mentioned psychopathological symptoms.

Taken altogether, chronic conditions such as oncological conditions, cardiological conditions, and diabetes represent a state of health that can determine an individual’s psychological vulnerability to CAS. Research on the role of meta-beliefs in the group of chronically ill people brings us closer to understanding the causes of the frequent incidence of depression and anxiety disorders in this section of the general population. It also helps us to discover new directions for psychotherapy as practical help for patients with chronic diseases who are at risk of higher mortality due to a deteriorated mental state. MCT is a justified idea for psychotherapeutic work in the groups of patients discussed above. The results of our research help to target specific elements that are worth considering when working with a particular patient group. An interesting continuation of the research would be to create a transdiagnostic MCT approach that would not only address the similarities between these diseases but also help to overcome the differences between them so that, in the future, people suffering from various chronic diseases could benefit from readily available psychotherapeutic groups built on the foundations of MCT. In this way, we could lower mortality rates among chronically ill patients—their condition can be, and frequently is, exacerbated by depressive and anxiety symptoms, which we could directly target.

## Figures and Tables

**Table 1:** Sociodemographic data in the studied group of patients, broken down by disease entities.

Sociodemographic	Cancer Patients %	Cardio Patients %	Diabetics %
Gender			
Male	28	72	57
Female	72	28	43
Age			
<25	0	3	24
26–45	20	13	41
46–60	30	23	27
>60	50	61	8
Education			
Primary	7	8	2
Vocational	15	40	22
Secondary	42	23	38
Higher	36	29	38
Marital status			
Single	9	8	38
Married	67	70	45
Divorced	10	5	12
Widowed	14	17	5
Residence			
Village	16	23	34
City < 50 k	22	20	24
City 50–100 k	17	10	16
City 100–500 k	14	45	9
City > 500 k	31	2	17

**Table 2:** Descriptive statistics and Pearson correlations for all variables observed in cancer patients.

Variable	M (SD)	1	2	3	4	5	6
HADS Anxiety	7.48 (3.05)						
2. HADS Depression	6.48 (2.91)	0.47					
3. MCQ-30 Positive beliefs	34.25 (10.2)	0.18	0.06				
4. MCQ-30 Negative beliefs	33.14 (9.59)	0.45 *	0.27	0.41			
5. MCQ-30 Cognitive confidence	18.55 (6.11)	0.19	0.14	0.4	0.71		
6. MCQ-30 Need to control thoughts	24.45 (6.48)	0.3 *	0.19	0.58	0.63	0.52	
7. MCQ-30 Cognitive self-consciousness	17.28 (3.92)	0.16	0.16	0.21	0.21	0.01	0.39

* *p* < 0.05.

**Table 3:** Descriptive statistics and Pearson correlations for all variables observed in cardio patients.

Variable	M (SD)	1	2	3	4	5	6
HADS Anxiety	8.5 (3.4)						
2. HADS Depression	6.43 (3.88)	0.52					
3. MCQ-30 Positive beliefs	39.65 (12.08)	0.29	0.22				
4. MCQ-30 Negative beliefs	33.63 (10.54)	0.45 *	0.46	0.7			
5. MCQ-30 Cognitive confidence	20.43 (5.48)	0.32 *	0.44	0.45	0.72		
6. MCQ-30 Need to control thoughts	27.35 (7.64)	0.27	0.25	0.85	0.79	0.52	
7. MCQ-30 Cognitive self-consciousness	17.63 (3.2)	0.31 *	0.32	0.72	0.53	0.39	0.7

* *p* < 0.05.

**Table 4:** Descriptive statistics and Pearson correlations for all variables observed in diabetic patients.

Variable	M (SD)	1	2	3	4	5	6
HADS Anxiety	7.59 (3.91)						
2. HADS Depression	5.23 (3.59)	0.62					
3. MCQ-30 Positive beliefs	35.22 (9.67)	0.46 *	0.41 *				
4. MCQ-30 Negative beliefs	32.16 (9.23)	0.69 *	0.52 *	0.6			
5. MCQ-30 Cognitive confidence	18.99 (5.59)	0.48 *	0.56 *	0.53	0.66		
6. MCQ-30 Need to control thoughts	25.95 (5.59)	0.38 *	0.33	0.64	0.7	0.64	
7. MCQ-30 Cognitive self-consciousness	18.17 (3.68)	0.43 *	0.26	0.6	0.65	0.42	0.67

* *p* < 0.05.

**Table 5:** Descriptive statistics and Pearson correlations for all variables observed in all studied chronically ill patients.

Variable	M (SD)	1	2	3	4	5	6
HADS Anxiety	7.93 (3.45)						
2. HADS Depression	6.08 (3.5)	0.55					
3. MCQ-30 Positive beliefs	35.54 (10.46)	0.35	0.26				
4. MCQ-30 Negative beliefs	32.77 (9.57)	0.57 *	0.43 *	0.54			
5. MCQ-30 Cognitive confidence	19.05 (5.84)	0.36	0.39 *	0.48	0.68		
6. MCQ-30 Need to control thoughts	25.46 (6.83)	0.35	0.27	0.66	0.68	0.58	
7. MCQ-30 Cognitive self-consciousness	17.72 (3.74)	0.32	0.22	0.46	0.47	0.26	0.57

* *p* < 0.05.

**Table 6:** Linear regression models predicting the severity of depressive and anxiety symptoms based on metacognitive beliefs.

Group	Variable (HADS Results)	Predictors (MCQ Scores)	Beta	t	F	p	R^2^
		Positive beliefs	0.31	1.41			
		Negative beliefs	1.98	7.12			
		Cognitive confidence	−0.07	−0.18			
		Need to control thoughts	−0.77	−1.91			
All patients	Anxiety	Cognitive self-consciousness	0.81	1.53	25.24	<0.001	0.322
		Positive beliefs	0.47	1.45			
		Negative beliefs	2.55	6.29			
		Cognitive confidence	0.76	1.28			
		Need to control thoughts	−1.52	−2.75			
	Anxiety	Cognitive self-consciousness	0.36	0.45	21.44	<0.001	0.488
		Positive beliefs	0.58	1.73			
		Negative beliefs	1.22	2.92			
		Cognitive confidence	2.34	3.83			
		Need to control thoughts	−1.14	−2.01			
T2DM patients	Depression	Cognitive self-consciousness	−0.69	−0.83	13.36	<0.001	0.366

## Data Availability

Dataset available on request from the authors.
